# NLStradamus: a simple Hidden Markov Model for nuclear localization signal prediction

**DOI:** 10.1186/1471-2105-10-202

**Published:** 2009-06-29

**Authors:** Alex N Nguyen Ba, Anastassia Pogoutse, Nicholas Provart, Alan M Moses

**Affiliations:** 1 Department of Cell & Systems Biology, University of Toronto, 25 Willcocks Street, Toronto, Canada; 2 Centre for the Analysis of Genome Evolution and Function, University of Toronto, 25 Willcocks Street, Toronto, Canada

## Abstract

**Background:**

Nuclear localization signals (NLSs) are stretches of residues within a protein that are important for the regulated nuclear import of the protein. Of the many import pathways that exist in yeast, the best characterized is termed the 'classical' NLS pathway. The classical NLS contains specific patterns of basic residues and computational methods have been designed to predict the location of these motifs on proteins. The consensus sequences, or patterns, for the other import pathways are less well-understood.

**Results:**

In this paper, we present an analysis of characterized NLSs in yeast, and find, despite the large number of nuclear import pathways, that NLSs seem to show similar patterns of amino acid residues. We test current prediction methods and observe a low true positive rate. We therefore suggest an approach using hidden Markov models (HMMs) to predict novel NLSs in proteins. We show that our method is able to consistently find 37% of the NLSs with a low false positive rate and that our method retains its true positive rate outside of the yeast data set used for the training parameters.

**Conclusion:**

Our implementation of this model, NLStradamus, is made available at:

## Background

Eukaryotic cells are defined by the presence of their nucleus. The nuclear membrane enclosing the genetic material of the cell is selective in its import of material through its nuclear pores and this translocation is mediated by cellular mechanisms [1,2].

Proteins entering the nucleus must do so through proteins forming the nuclear pores: the nuclear pore complex [3,4]. The pores allow the passive diffusion of small proteins, but bigger proteins entering the nucleus are usually bound by karyopherin complexes on their nuclear localization signal [5]. Although there are many nuclear import pathways in eukaryotic cells, most of these have not been characterized in detail. The best understood is the classical NLS pathway. The recognition of classical NLSs on nuclear proteins is done by the importin-α subunit which in turn is recognized by the importin-β subunit. This trimer (cargo, importin-α and importin-β) is then imported to the nucleus after series of enzymatic steps [1,6]. Other families of NLSs are independent of importin-α, and may bind directly to one of the members of the importin-β superfamily [1].

Classical NLSs show characteristic patterns of basic residues loosely matching two consensus sequences, K(K/R)X(K/R) and KRX_10–12_KRXK, termed the 'monopartite' and 'bipartite' classical NLS [1,2]. PSORT [7] accurately predicts protein localization by including heuristic scores based on residues frequencies, protein domains as well as motifs. PSORT will then offer, based on the cNLS consensus sequences, various possible NLSs on nuclear proteins.

However, many known NLSs do not match any of the consensus sequences described above. Therefore, other computational methods have been proposed to predict NLSs based on amino acid sequences. One method, PredictNLS [8], employs a database of regular expressions to predict the various types of NLSs.

Here we sought to test the power of these methods to predict NLSs in *Saccharomyces cerevisiae*. We compiled a list of experimentally verified NLSs in yeast and found that PredictNLS [8] predicts 10% of the NLSs in this set. Based on an analysis of the residues frequencies in the NLSs in this set, we developed a simple hidden Markov model [9] that can be used to predict the nuclear localization signal in proteins, achieving true positive rate of 37%. We also explored using specific models for the monopartite and bipartite NLSs, but surprisingly, found no significant improvement in positive predictive value. Finally, we also show that our method fares well in species other than yeast, consistent with the conservation of import mechanisms.

## Results

### A set of experimentally verified nuclear localization signals in yeast

In order to test the predictive power of computational methods to predict NLSs in *S. cerevisiae*, we sought to identify a set of experimentally confirmed NLSs. We searched the literature for papers in which specific stretches of amino acids have been shown to act as functional NLSs in yeast. While many approaches have been used to characterize and identify nuclear localization signals in proteins, we took as the NLS the minimal sequence in the protein that was proven to be either necessary or sufficient for nuclear import.

To categorize NLSs into their respective import pathways, we looked for evidence of their respective pathways which includes receptor binding or pathway dependency. However, only half of our proteins had a clear reference to these, and many remain unknown.

In all, we found 60 NLSs. Of these, 16 had evidence of importin-α dependence or binding and we here refer to these as cNLSs. In addition, we found 15 NLSs which depend on other import pathways, which we term non-cNLSs. Finally, for 29 of our sequences, we failed to find information about the mechanism of import. The NLSs and references are listed in Tables 1 and 2.

**Table 1:** non-cNLS and cNLS sets

ORF	Gene name	Start	Stop	Necessity or Sufficiency (PMID)	Receptor binding or pathway dependency (PMID)
YBR009C	HHF1	4	21	11694505	11694505^b^
YBR010W	HHT1	10	28	11694505	11694505^b^
YDL007W	RPT2	11	15	15210724	15210724^a^
YDL007W	RPT2	33	37	15210724	15210724^a^
YDR103W	STE5	49	66	10481914	10481914^b^
YDR146C	SWI5	636	655	7615496/1652372	18485366^a^
YDR208W	MSS4	347	364	12912920	12912920^b^
YDR224C	HTB1	30	36	3123916	15679097^b^
YEL009C	GCN4	231	246	12455686	18485366^a^
YER040W	GLN3	388	394	12624103	12624103/18485366^a^
YFR034C	PHO4	141	166	9732266	9732266^b^
YGL071W	AFT1	202	207	14523005	14523005^b^
YGL071W	AFT1	352	355	14523005	14523005^b^
YGL071W	AFT1	332	335	14523005	14523005^b^
YGL097W	SRM1	3	23	18485366	18485366^a^
YHR079C	IRE1	645	657	17035634	17035634^a^
YIL075C	RPN2	811	832	15210724	15210724^a^
YIL150C	MCM10	512	527	13680157	18984568^a^
YIL150C	MCM10	435	451	13680157	18984568^a^
YJL194W	CDC6	27	33	18485366	18485366^a^
YLR103C	CDC45	209	228	18485366	18485366/18984568^a^
YLR182W	SWI6	157	169	14998990	14998990^a^
YML007W	YAP1	5	59	11274141	11274141^b^
YMR127C	SAS2	19	35	15788653	15788653^b^
YMR239C	RNT1	461	466	15090619	15337846^a^
YNL027W	CRZ1	394	422	11535618	11535618^b^
YNL027W	CRZ1	612	615	11535618	11535618^b^
YOL123W	HRP1	522	534	18343812	18343812^b^
YOL127W	RPL25	18	28	1920406	9182759/9687515^b^
YOL127W	RPL25	11	17	1920406	9182759/9687515^b^
YPL153C	RAD53	785	807	15972895	15972895^a^
YPR119W	CLB2	183	200	18485366	18485366^a^

Characterized yeast nuclear localization signals used in our analysis for which import pathway is known. ^a ^corresponds to classical NLSs, ^b ^corresponds NLSs known not to dependent on the classical NLS import pathway. See text for details of how NLSs were classified. Numbers in the last two columns indicate references using pubmed IDs (PMID).

**Table 2:** unknown NLS set

ORF	Gene name	Start	Stop	Necessity or Sufficiency (PMID)
YAL040C	CLN3	559	580	11509671
YBL105C	PKC1	810	813	15643058
YBR098W	MMS4	244	263	14642571
YCL017C	NFS1	312	316	11110795
YCL067C	HMLALPHA2	1	13	1976249
YCL067C	HMLALPHA2	141	159	1976249
YCR039C	MATALPHA2	2	13	8757785
YCR039C	MATALPHA2	141	159	8757785
YDR034C	LYS14	190	250	10975256
YEL032W	MCM3	766	772	16093348
YEL061C	CIN8	994	1000	11694576
YGL103W	RPL28	24	30	2104804
YGL103W	RPL28	7	14	2104804
YGR027C	RPS25A	11	36	10386617
YGR027C	RPS25A	87	95	10386617
YIR006C	PAN1	1024	1040	17967424
YIR006C	PAN1	1145	1161	17967424
YJL157C	FAR1	11	30	10485850
YJL157C	FAR1	38	48	10485850
YJL187C	SWE1	304	310	18562688
YJL190C	RPS22A	21	29	10386617
YKL112W	ABF1	624	628	15522095
YLR079W	SIC1	77	89	16294029
YML024W	RPS17A	2	7	3939318
YMR036C	MIH1	31	33	18562688
YOR063W	RPL3	1	21	3931077
YOR274W	MOD5	408	424	9872948
YPR189W	SKI3	306	314	2660461

Characterized yeast nuclear localization signals used in our analysis, for which we could not find evidence for a specific import pathway. Numbers in the last column are pubmed IDs for references. See text for details of how NLSs were classified.

### Current NLS prediction methods show little predictive power on our yeast data

**Figure 1:**
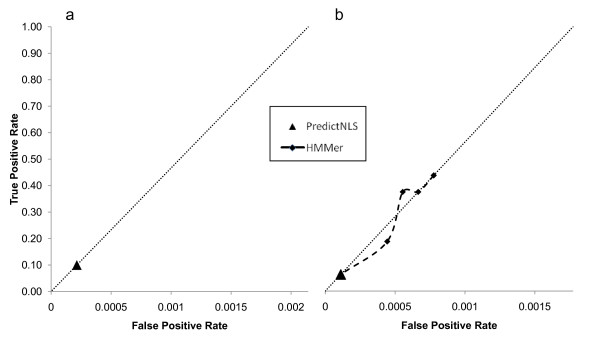
**True positive and false positive rate of consensus and alignments based methods**. a) True positive and false positive rate of a consensus-based method on all NLSs from our dataset. The false positive rate is shown as the error rate per amino acid residue. The diagonal line depicts a ratio of one true prediction per false prediction per amino acid residue. b) True positive and false positive rate of consensus and alignment based methods on classical NLSs from our dataset. The false positive rate is shown as the error rate per amino acid residue. The diagonal line depicts a ratio of one true prediction per false prediction per amino acid residue.

In order to test the power of NLS prediction methods, we first used the consensus sequence-based approach PredictNLS [8] and found that it was generally too specific: only 10% (6/60) of our characterized NLSs were identified by their genome wide analysis of the yeast proteome (see Figure 1a). These results were surprising, considering that this method had been reported to find 100% of the experimentally characterized NLSs considered in the general study [8]. We suggest that PredictNLS might be underestimating the true variability of NLSs, as few yeast NLSs (9) were included in that study. PredictNLS also produced 6 predictions that were not characterized NLSs, suggesting a positive predictive value of 50%.

Probabilistic models have been widely used to predict domains in proteins (E.g., Pfam [10]). Profile HMMs model a linear series of states which approximately corresponds to the pattern of residues in a consensus motif. These models can account for length variation using insertion and deletion states.

**Figure 2:**
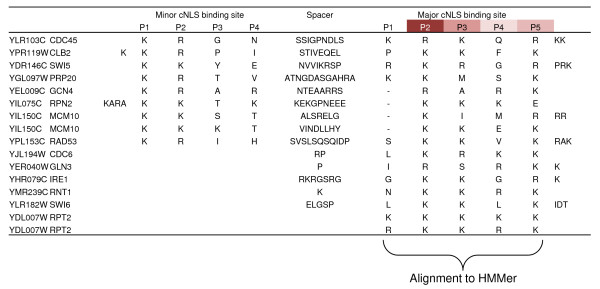
**Alignment of characterized classical nuclear localization signals**. Alignment of the residues thought to contribute to NLS binding to importin-α. The residues aligned on the cNLS major binding site were then used as model for a profile HMM approach using HMMer.

NLSs with different import mechanisms are unlikely to share a consensus motif. Therefore, we tried a profile HMM approach to predict only cNLSs. We manually aligned the labelled cNLSs using a proposed biological model of binding specificity of importin-α [9] (Figure 2) and built an HMM model using HMMbuild [11] (see Methods). We used this model to predict cNLSs using HMMsearch, and assessed the predictive power at different E-value thresholds using a leave-one-out cross-validation (see Methods). The results are displayed as ROC curves in Figure 1b (see Methods) and indicate that HMMer obtains a similar positive predictive value (PPV) as PredictNLS at varying true positive rate (TPR).

Taken together, our results indicate that both frameworks show some positive predictive value but PredictNLS shows a low true positive rate, and both methods show PPV of around 50% in our data.

### The nuclear localization signal shows a strong statistical difference in residue frequencies

While the NLSs can be divided in many functional categories depending on their pathways, we observed that most NLSs had an enrichment of basic residues. We first analyzed the residue content of the characterized cNLSs. Consistent with the definition of classical NLSs, we observe significant frequency differences (see Methods) mainly in lysine (7.3% for the genome vs 29.3% for the cNLSs, P-value < 10^-10^) and arginine (4.4% for the genome vs 15.7% for the cNLSs, P-value < 10^-10^).

To determine whether there were differences between the different types of NLSs, We then compared the set of cNLS to all other NLS (non-cNLS and unknown) and surprisingly found no significant differences in their residue frequencies. Thinking that maybe the unknown set of NLSs might have been mostly composed of cNLSs, we also compared the cNLS residue frequencies to the non-cNLS residue frequencies and also found no significant differences in residue frequencies. Thus, in our data set the three categories of NLSs (cNLS, non-cNLS and unknown) show no difference in residue frequencies.

**Figure 3:**
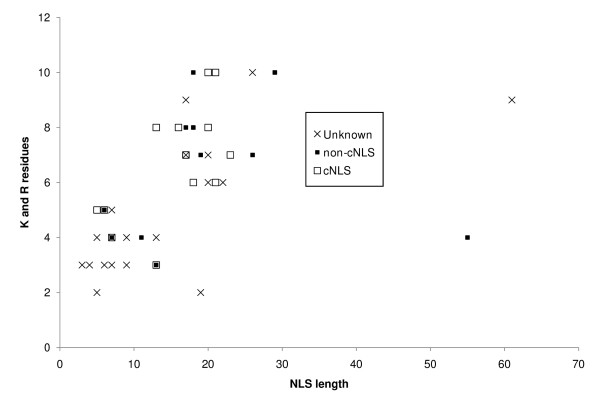
**Lysine and arginine content of characterized nuclear localization signals**. Plot of the lysine and arginine content of characterized nuclear localization signals with respect to their length. The plot shows the three 'types' of NLSs present in our study.

Because the most apparent statistical difference lies in the frequencies of lysine (K) and arginine (R), we plotted each NLS in respect to number of K and R residues and their length (Figure 3), and consistent with the previous residue frequencies test, we found that there were generally no discernable differences between the three categories of NLSs.

Interestingly, we also observe two groups of NLSs on this plot, the first showing an average length of 8 amino acids containing an average of 3.9 basic residues while the other showing an average length of 20 amino acids containing an average of 7.3 basic residues. We suggest that these regions correspond to monopartite and bipartite NLSs [1]. We note that even NLSs known not to be importin-α dependent showed this pattern.

### A simple Hidden Markov Model shows better predictive performance

**Figure 4:**
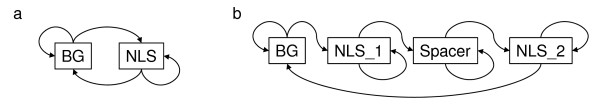
**Schematic of our two state and four state HMM**. a) The two state HMM models two states which are represented by the 'background', which emits residues with the same frequency as the genome, and by the 'NLS' state, which emits residues with the same frequency as the NLSs from our characterized data. b) The four state HMM models four states which are represented by the 'background', which emits residues with the same frequency as the genome, two 'NLS' states, which emit residues with the same frequency as our characterized NLSs, separated by a 'spacer' state which emits residues with the same frequency as the genome.

**Figure 5:**
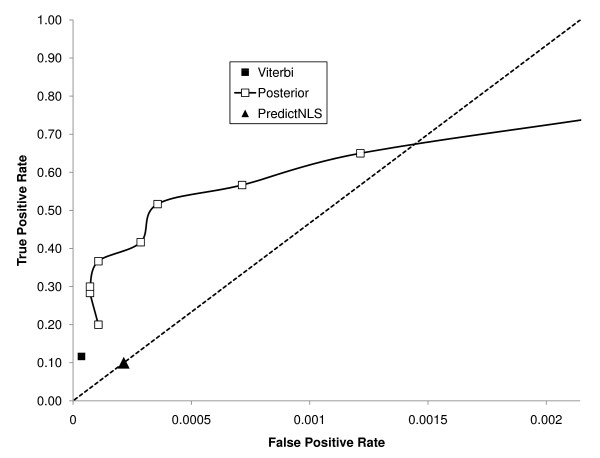
**True positive and false positive rate of our model**. True positive and false positive rate of various methods, including our HMM at various posterior threshold and the Viterbi algorithm on our dataset. The false positive rate is shown as the error rate per amino acid residue. The diagonal line depicts a ratio of one true prediction per false prediction per amino acid residue.

Motivated by the idea that all NLSs in our set shared a similar bias in lysines and arginines, we created a simple two-state HMM. In this model, sequence is generated either from a background model (with residue frequencies equal to those in the genome) or an NLS model, whose residue frequencies are equal to those in all of the characterized yeast NLSs (Figure 4a). To assess the positive predictive value of the model, we performed a leave-one-out cross validation, as above (see Methods), of the HMM. We assessed its predictive strength in two ways, either using the most probable path (the Viterbi algorithm) or by computing the posterior probability and counting predictions when it passed a certain threshold (see Methods). Relative to PredictNLS, the results show significantly improved positive predictive value (PPV = 88% vs. PPV = 50%, P-value < 0.05), and higher true positive rate (TPR = 37% vs. TPR = 10%, P-value < 0.001, Figure 5) while producing only 3 false positives. Our results were specific to the experimentally defined NLSs. We defined a residue-level correlation coefficient (analogous to nucleotide level correlation coefficient [12,13] for DNA motifs) and found that our model yielded a coefficient of 0.36. We also computed the Matthews Correlation Coefficient [14] and found that it was 0.55, similar to recent results for a predictor of nuclear export signals [15].

We were surprised to observe that a single, simple model could achieve increased performance even though our training data spanned a large diversity of NLSs, e.g., some cNLSs and others not, both bipartite and monopartite. Nevertheless, we found that our method showed similar positive predictive value on each set of NLSs when we analyzed them individually (data not shown). While perhaps surprising, these results are consistent with the similar residue compositions of each sets of NLSs (Figure 3).

### A more complex model does not improve prediction

**Figure 6:**
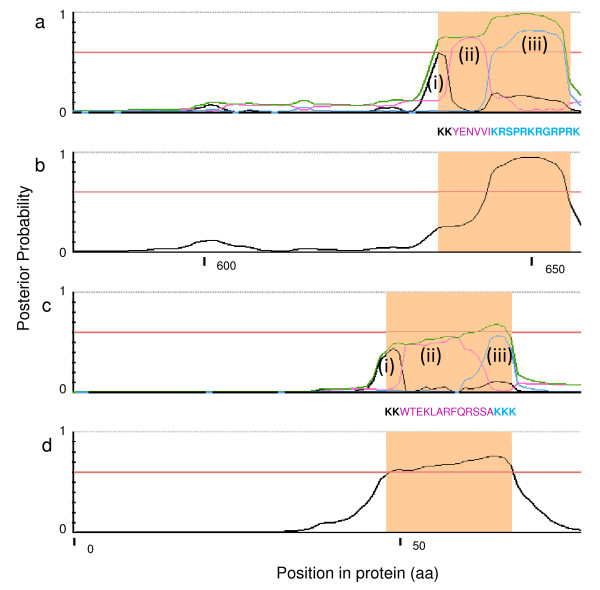
**Posterior trace of Swi5p and Ste5p for our two HMMs**. a) Posterior trace of Swi5p, a characterized bipartite cNLS, using our four state model. Output was generated by NLStradamus and highlighted region shows the region of characterized NLS. Black (i) and blue (iii) lines represent the two patches of basic residues while the pink line (ii) represents the spacer. Green line represents the sum of the three NLS states. Red line is shown as a reference for a threshold of 0.6. b) Posterior trace of Swi5p, a characterized bipartite NLS, using our simple two state model. Output was generated by NLStradamus and highlighted region shows the region of characterized NLS. Horizontal red line depicts the chosen posterior threshold of 0.6. c) Posterior trace of Ste5p, a characterized bipartite importin-β dependent NLS (non-cNLS), using our four state model. Output was generated by NLStradamus and highlighted region shows the region of characterized NLS. Black (i) and blue (iii) lines represent the two patches of basic residues while the pink line (ii) represents the spacer. Green line represents the sum of the three NLS states. Red line is shown as a reference for a threshold of 0.6. d) Posterior trace of Ste5p, a characterized bipartite non-classical NLS, using our simple two state model. Output was generated by NLStradamus and highlighted region shows the region of characterized NLS. Horizontal red line depicts the chosen posterior threshold of 0.6.

We observed two main regions within our K-R content plot where NLSs seemed to aggregate and this is consistent for the bipartite and monopartite classes of cNLSs, but we were surprised to see that this was also observed in the other sets. To test whether or not we could improve our predictions using this information, we created a four state HMM by modelling a 'spacer' state between two patches of basic residues (Figure 4b, see methods) and analyzed its predictive power using a leave-one-out cross-validation. This model is able to recognize the two basic stretches of the NLS separated by a spacer region, which we model using the background residue frequencies. We refer to this model as the 'bipartite model'. In some cases, this model accurately identifies the boundaries of these regions (Figure 6a, c). For comparison, we also trained a model using only the shorter NLSs, which we refer to as the 'monopartite model'. We refer to the initial model that uses the frequency of all of the NLSs as the 'combined model'.

To test these models, we defined bipartite and monopartite NLSs based on the two classes we had observed in Figure 3. We tested the bipartite model on the bipartite NLSs only. Surprisingly, the positive predictive value and true positive rate of the bipartite model was exactly the same as the combined model (TPR = 61%, same FPR), as the combined model can also identify bipartite NLSs (Figure 6b, d). Similar predictive power was also observed when comparing the monopartite model to the combined model on only monopartite NLSs. However, a small increase in true positive rate was observed for the monopartite model (TPR = 41% vs 38%, same FPR) but this difference was not significant.

### Applying the model to other organisms

**Figure 7:**
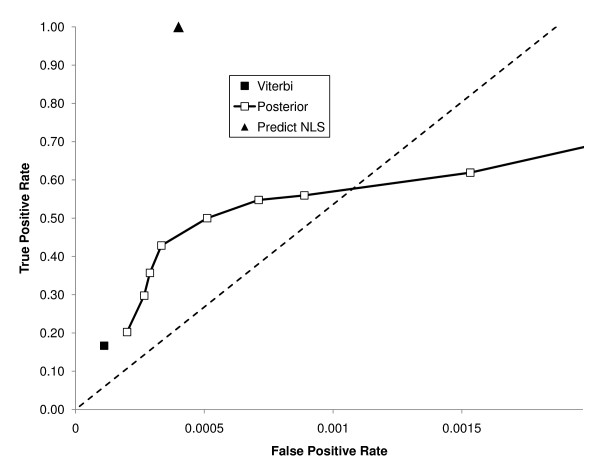
**True positive and false positive rate of our model on other species**. True positive and false positive rate of various methods, including our HMM at various posterior threshold and the Viterbi algorithm on the PredictNLS dataset. This ROC curve was created by counting overlaps. The false positive rate is shown as the error rate per amino acid residue. The diagonal line depicts a ratio of one true prediction per false prediction per amino acid residue.

We were concerned that our predictor might have a bias for yeast proteins. We therefore tested our method on the set of NLSs used to train PredictNLS [8]. This set of data contains NLSs that have been shown to function in vertebrate cells. We found that our method has a similar true positive rate in this data as it does in yeast, finding 37% of the characterized NLSs at the same posterior threshold. However we do note an increased false positive rate (228% increase, P-value = 0.08), and modest reductions in the nucleotide-level correlation coefficient to 0.30 (from 0.36, Figure 7) and the Matthews Correlation Coefficient to 0.48 (from 0.55).

Seeking to understand the elevated rate of false predictions, we explored them further and found that 7 of the 15 vertebrate false positives occur on only three proteins. When we searched the literature for more information on these proteins we found that one of our 'false' predictions had actually been identified as an NLS [16,17]. We identified on another protein two patches of amino-acid repeats, and finally on the last protein two nucleolar localization signals, which are long stretches of basic amino acid residues, and it is not yet clear if these signals should be considered as NLSs [18,19].

We noted in total three false predictions composed of amino-acid repeats, which were largely absent from our yeast training set. Such repeats are problematic because they violate an assumption of our simple hidden-Markov model, namely, that adjacent residues are independent given the value of the hidden state.

Taken together, this analysis of vertebrate NLSs demonstrates that the true positive rate of our method is not confined to our training data set. However, we did note an increase in false positives, which might be explained by additional undiscovered NLSs in this data set, or repetitive sequences in vertebrate proteins.

## Discussion

Analysis of residue frequency clearly shows that there is a bias in key residues in the NLS, and while previous studies have shown that there exists some position requirement in these residues [20], the NLSs do not clearly align and therefore we believe that the signal simply do not obey a clear consensus sequence rule. Our results are consistent with the model that NLSs may simply be regions of high positive charges with only minor spatial requirements [20]. This idea is consistent with the model that the NLS can be masked by phosphorylation [21], by inhibiting the activity of the signal due to addition of negative charges.

There is abundant evidence that cNLSs bind at specific positions on importin-α and this binding is mediated by two sites which bind monopartite or bipartite cNLSs [22,23], which suggests that the bipartite cNLS is not simply a bigger monopartite cNLS. We were, however, unable to exploit this knowledge to create a stronger predictor. While each basic patch in the bipartite cNLS does not represent a monopartite cNLS, the combination of both patches is sufficient for recognition by our predictor.

By examining some false negatives, we find at least two reasons why our method fails to predict them. First, there are examples that do not seem to show an enrichment of basic residues. Second, other NLSs are simply too small to be identified reliably, due to a lack of statistical signal.

We note that unlike PSORT which is based on the cNLS consensus motifs, our method does not attempt to predict NLSs belonging to a particular pathway. We observed that all NLSs show an excess of basic residues, and therefore we have developed a simple HMM that can identify stretches of basic residues in protein sequences. It is somewhat surprising that this method performs reasonably well. This suggests that the consensus patterns for different import pathways may be more similar than currently anticipated.

We believe that obtaining a higher positive predictive value is certainly possible by combining other biological knowledge; however, this is still under study. For example, some of our false predictions might either be real NLSs that have not yet been characterized, or simply part of other undiscovered protein domains.

## Conclusion

In conclusion, we offer a simplified approach to predicting nuclear localization signals and show that this method can be applied to multiple species. This agrees with the notion that important regulatory mechanisms are conserved, although it is possible that differences in positive predictive value can be attributed to the existence of multiple importin-α,β homologues in other species [24] or differences in proteins lengths between species.

## Methods

### Creation of alignments and assessment of consensus sequence based methods

Based on recent studies on the binding sites of the importin-α [9], we created an alignment of our cNLS based on the major site of the importin (Figure 2). This alignment was run through HMMer [11] using HMMbuild and calibrated using HMMcalibrate. Using HMMsearch and a leave-one-out cross-validation [25], we assessed its predictive power. The leave-one-out cross-validation of the HMMer framework was done by removing all the characterized NLSs of one of the proteins from the alignment and then applying the above method on the protein.

### Analysis of residue frequency in the nuclear localization signal and statistical significance

We first verified if the NLS containing proteins had different residue frequencies than the genome to verify if our proteins were different than expected in the genome and found that this was the case. We used a sampling analysis (data not shown) and a two-tailed Fisher's exact test. We computed



where *a*_*ij *_is the number of counts in *i*^th ^row and *j*^th ^column of a 2 × 2 table, *R*_1_, *R*_2_, *C*_1 _and *C*_2 _are the row and column totals, respectively, and *N *is the total number of observations. To calculate the two-tailed P-value using Fisher's exact test, we summed the probabilities of all the matrices below and equal to the *P*_*cutoff*_. If the P-value was below than 0.01 we considered that residue significant.

### Creation of a Hidden Markov model

We created a simple two state HMM as well as a four state HMM to assess the difference between monopartite and bipartite motifs. The two state HMM receives two sets of states based on the background and NLS residue frequencies, while the four state HMM assumes that the spacer residue frequencies are equal to the 'background' residue frequencies. A four state HMM where the spacer residue frequencies were taken directly from our characterized data was also done (data not shown). Transition probabilities were approximated by summing the number of times that a transition occurs divided by the total amount of transitions from a given state in our data set. The starting state was assumed to be the same as the 'background' state. Further analysis using the Baum-Welch algorithm [26,27] was used but did not yield significant predictive power difference. Outputs are both the most probable path and posterior probabilities. This HMM is publically accessible from our web page.

The transition probabilities between each state were calculated as:



Where a_kl _is the transition probability from state k to state l and A_kl _is the observed frequency of this transition in the characterized data.

The posterior probability at a given position was calculated as in [26]:



The posterior probability is the probability that the i^th ^amino acid in the given sequence (x) is produced by state k (π_i _= k). This is calculated using the forward algorithm, which is the probability of all the sequences up to and including the i^th ^amino acid requiring that the i^th ^amino acid is produced by state k (f_k_(i)), multiplied by probability of all sequences after the i^th ^amino acid when the i^th^amino acid is produced by state k (b_k_(i)), given by the backward algorithm, and divided by the probability of the whole sequence given the model (P(x)).

### Signal detection, optimizing true positive rate and ROC curve

We used several thresholds on the posterior probability and any segment over the threshold is labelled as a signal for the two state model. The four state model instead sums the two NLS states as well as the spacer state and establishes a signal when this sum is over the threshold. Using signal/true motif overlaps, we assess the true positive rate and positive predictive value using a leave-one-out cross-validation [25]. The leave-one-out cross validation was done systematically by removing a protein from the training set, and testing the predictor on this protein.

The threshold whose ratio is furthest from a ratio of one true positive prediction per false prediction was deemed to be the strongest. This ratio, one true positive per false prediction, is shown in our ROC curves as a dotted diagonal line. We note that this diagonal is not indicative of a random predictor as in traditional ROC curves. We also calculated a residue correlation coefficient (rCC) [12] to assess performance of the model.



The rCC is the Pearson product-moment coefficient of correlation, where TP and TN are the number of true positives and true negatives, while FP and FN are the number of false positives and false negatives, taken at the nucleotide level as opposed to the signal level. Because our predictions are done on protein sequences, this was done at the residue level instead of nucleotides as is done for DNA motifs [12].

A Matthews' correlation coefficient is calculated similarly to the calculation of the rCC, however this is taken at the signal level and taking as the number of true negatives the number of predictions made at a threshold of 0.001. This is a similar method as used by predictNES [14,15]. This number of true negatives was also used to assess significance of false positive rate differences in a Fisher's test as above.

True positive rate (TPR) is calculated as the number of true predictions divided by the number of total possible true positives, while the false positive rate (FPR) is calculated as the number of false positives divided by the sum of the length of all our characterized proteins. The false positive rate is therefore given at the residue level, while the true positive rate is given at the level of individual NLSs. Positive predictive value (PPV) is assessed as the ratio of true positive predicted NLSs to the total number of predicted NLSs.

## Availability

The HMM-based prediction method, NLStradamus, for predicting nuclear localization signals can be accessed directly at: . Both two state and four state HMMs are available for use and posterior decoding at different thresholds is also possible.

## Authors' contributions

ANNB wrote the software, designed and performed the experiments, identified characterized motifs and wrote the paper. AP identified characterized motifs. NP provided insights on possible displays for the web software and edited the manuscript. AMM wrote the paper and designed the experiments. All authors have read and approved the final manuscript.
